# The composition of demand for newly launched vaccines: results from the pneumococcal and rotavirus vaccine introductions in Ethiopia and Malawi

**DOI:** 10.1093/heapol/czv103

**Published:** 2016-02-07

**Authors:** B Adam Williams, Teklay Kidane, Geoffrey Chirwa, Neghist Tesfaye, Marta R Prescott, Soleine T Scotney, Moussa Valle, Sintayehu Abebe, Adija Tambuli, Bridget Malewezi, Tahir Mohammed, Emily Kobayashi, Emily Wootton, Renee Wong, Rahima Dosani, Hamsa Subramaniam, Jessica Joseph, Elif Yavuz, Aliza Apple, Yann Le Tallec, Alice Kang’ethe

**Affiliations:** ^1^Clinton Health Access Initiative (CHAI) the global headquarters: 383 Dorchester Avenue, Suite 400, Boston, MA 02127 USA,; ^2^Ministry of Health, Maternal and Child Health Division, P.O. Box 30377, Lilongwe 3 Malawi and; ^3^Ministry of Health, Maternal and Child Health Division, Lideta Subcity Addis Ababa, Ethiopia P.O. Box 1234

**Keywords:** Decision-making, developing countries, diarrhoea, effectiveness, evidence-based policy, health care utilization, health planning, immunization, maternal and child health, policy implementation

## Abstract

Understanding post-launch demand for new vaccines can help countries maximize the benefits of immunization programmes. In particular, low- and middle-income countries (LMICs) should ensure adequate resource planning with regards to stock consumption and service delivery for new vaccines, whereas global suppliers must produce enough vaccines to meet demand. If a country underestimates the number of children seeking vaccination, a stock-out of commodities will create missed opportunities for saving lives. We describe the post-launch demand for the first dose of pneumococcal conjugate vaccine (PCV1) in Ethiopia and Malawi and the first dose of rotavirus vaccine (Rota1) in Malawi, with focus on the new birth cohort and the ‘backlog cohort’, comprised of older children who are still eligible for vaccination at the time of launch. PCV1 and Rota1 uptake were compared with the demand for the first dose of pentavalent vaccine (Penta1), a routine immunization that targets the same age group and immunization schedule. In the first year, the total demand for PCV1 was 37% greater than that of Penta1 in Ethiopia and 59% greater in Malawi. In the first 6 months, the demand of Rota1 was only 5.9% greater than Penta1 demand in Malawi. Over the first three post-introduction months, 70.7% of PCV1 demand in Ethiopia and 71.5% of demand in Malawi came from children in the backlog cohort, whereas only 28.0% of Rota1 demand in Malawi was from the backlog cohort. The composition of demand was impacted by time elapsed since vaccine introduction and age restrictions. Evidence suggests that countries’ plans should account for the impact of backlog demand, especially in the first 3 months post-introduction. LMICs should request for higher stock volumes when compared with routine needs, plan social mobilization activities to reach the backlog cohort and allocate human resources and cold chain capacity to accommodate high demand following vaccine introduction.

Key Messages

- Understanding post-launch demand for new vaccines can help countries maximize the benefits of immunization programmes. When planning a new vaccine introduction, countries should consider the impact of the ‘backlog cohort’ of age-eligible children born before the introduction of the vaccine, especially in the first 3 months of introduction.
- In the case of Ethiopia and Malawi, in the first year of pneumococcal conjugate vaccine (PCV) introduction, the demand for the new vaccine (PCV1) was 37% and 59%, respectively, greater than that of the benchmark vaccine (Penta 1), mostly due to demand from older ‘backlog’ children.
- Countries can maximize the life-saving potential of immunization through vaccinating a larger share of the ‘backlog cohort’ by setting looser age eligibility criteria.
- To cater to this backlog cohort, countries’ plans should request high stock volumes for the new vaccines, allocate adequate human resources and cold chain capacity to accommodate backlog cohort demand, and include social mobilization activities to reach more children in the backlog cohort and reduce drop-out.

## Introduction

A significant wave of new vaccine introductions (NVIs) is taking place worldwide. For instance, 124 countries have already introduced the pneumococcal conjugate vaccine (PCV), whereas 25 countries are planning its introduction (IVAC 2015). Seventy-seven countries have introduced the rotavirus vaccine (Rota), whereas 27 countries are planning its addition to their routine immunization programme (IVAC 2015). This acceleration follows the ever-increasing pace of new vaccine development (Moxon and Siegrist 2011) and efforts of the global community to encourage low- and/or middle-income countries (LMICs) to adopt new vaccines (Andrus *et al.* 2007; Widdowson *et al.* 2009; Hajjeh *et al.* 2010).

Although recent immunization research has focused on analysing factors leading to the decision to introduce a new vaccine (Wenger *et al.* 1999; Andrus *et al.* 2011; Munira and Fritzen 2007; Levine *et al.* 2011; Burchett *et al.* 2012; Makinen *et al.* 2012), there is a need for increased evidence on *how* to best introduce these new vaccines.

In LMICs, the current vaccine introduction process typically consists of several steps. First, when developing a new comprehensive multi-year plan for immunization (cMYP) every 5 years, Ministries of Health and their partners complete a situational analysis of the national epidemiological context and immunization system. From this assessment, countries identify the new vaccines that should be included in their national programme, and define tentative dates for introduction as part of the multi-year workplan. Currently Gavi-eligible countries submit an expression of interest, followed by an application for support to Gavi, the Vaccine Alliance. Indeed, availability of Gavi funding is one of the main determinants of countries’ deciding to introduce a vaccine (Burchett *et al.* 2012). As part of their proposals to Gavi, countries define the number of children that will be targeted for the new vaccine in each year of the cMYP’s remaining validity. The support provided by Gavi follows recommendations from the World Health Organization (WHO) and The Strategic Advisory Group of Experts (SAGE) on Immunization, as described in WHO’s position papers on vaccines (WHO 2015). If the country’s application receives official approval through the Gavi review process—outlined in greater detail in Gavi’s general guidelines for expressions of interest and applications (Gavi 2015a)—the country will receive the Vaccine Introduction Grant (VIG) and procure vaccine and devices by the country or one of Gavi’s procurement agencies. The national Expanded Programme on Immunization (EPI) then decides on the exact day of introduction and carefully executes the preparatory activities. Approximately 6–12 months after introduction, a Post-Introduction-Evaluation is organized by EPI and partners to assess and course-correct issues with the roll-out of the vaccine.

One difficult decision at the time of the Gavi application is the forecast of demand for the vaccine: countries must estimate how many children will receive the first and last dose of the vaccine during each year of the programme for the cMYP period (Gavi 2010a,b). Characterizing the likely demand for a new vaccine over the year of introduction helps inform programmatic planning for better allocation of the limited vaccine stock, human resources and cold chain capacity, thus maximizing the health impact of the new vaccine.

Today, supply forecasting exercises for NVI typically estimate future consumption based on the number of age-eligible children born in the year after introduction (i.e. the new birth cohort) as the demand, and factor in wastage and a buffer stock to estimate the number of doses received in Year 1. However, the actual demand for vaccines may also include a ‘backlog cohort’, the older children who are eligible for vaccination at the time of introduction. For instance, the routine immunization schedules in Ethiopia and in Malawi stipulate that children be vaccinated with the first dose of PCV at 6 weeks of age, but children remain eligible for this vaccine until their first birthday. Thus, children younger than 6 weeks at the time of introduction and those born in the year after introduction are part of the ‘new birth cohort’. But children between 6 weeks and 1 year of age at the time of introduction may also seek vaccination, and these children constitute the backlog cohort.

Neither Gavi guidelines nor the cMYP costing tool guidance (WHO 2013) refer to the need to plan for the inclusion of a backlog cohort in the demand forecast for the new vaccine. For instance, in part due to supply constraints, Gavi guidance on PCV applications (Gavi 2015b) mentions that ‘Gavi does not provide support for catch up campaigns for pneumococcal vaccines’. Moreover, Gavi’s VIG is calculated based on the estimated size of the new birth cohort in the year of introduction (e.g. US $0.80 per infant in the new birth cohort or $100 000—whichever is greatest; Gavi 2015a). Thus, the proposals for PCV and Rota from Malawi, and for PCV from Ethiopia, initially estimated the demand for the vaccine purely based on the new birth cohort (Gavi 2010a,b).

Where age eligibility rules have been chosen based on a careful understanding of medical benefits, countries can generally maximize the life-saving potential of immunization by vaccinating a larger share of the backlog cohort. This is true if vaccination elicits seroconversion—and thus protection against disease—in older children within the backlog cohort before natural infection occurs. In addition, the risk of disease acquisition must continue after the date of the last scheduled dose, as was the case in Ethiopia and Malawi. Indeed, 31% of all hospitalized WHO clinical pneumonia cases in Africa occurred in children aged 12–23 months (Russell *et al.* 2011), much later than Week 14, the age of the last PCV dose in the Ethiopia and Malawi immunization schedules. In Malawi, hospitalizations for rotavirus-related gastroenteritis peak at 26 weeks (Sanderson *et al.* 2011), much later than the last scheduled rotavirus vaccine dose at Week 10. In addition, ‘herd protection’ of the unvaccinated occurs when a sufficient proportion of the group at risk is immune (WHO 2014b). Limiting vaccination to the new birth cohort may make it more difficult to quickly reach herd immunity, as the backlog cohort would continue to be at risk of disease transmission. Thus, where clinically relevant as in Ethiopia and Malawi, wider age eligibility bands, which lead to a larger backlog cohort receiving vaccination, offer an opportunity to maximize the benefits of immunization.

Once age eligibility rules are set, failure to incorporate the backlog cohort demand into NVI planning can lead to suboptimal outcomes. For instance, if a country underestimates the consumption of a new vaccine by the backlog cohort, the introduction of the new vaccine will yield partially vaccinated or unvaccinated children due to stock-outs or rationing of the new vaccines.

There is a paucity of evidence on the quantitative composition of demand between new and backlog cohorts. In particular, no published articles have until now conducted analysis of health facility-level child vaccination records to understand uptake dynamics over the first year of a vaccine introduction. As a result, little guidance exists on the backlog cohort’s impact on stock and routine immunization administration in the introduction year.

This study offers a novel empirical comparison of three vaccine introductions. We are able to compare the introduction of two different vaccines (three-dose PCV13 and two-dose Rota) in Malawi, and compare the introduction of the same vaccine (three-dose PCV13 and three-dose PCV10) in two different countries (Malawi and Ethiopia, respectively). The Clinton Health Access Initiative (CHAI) collaborated with Ministries of Health in Ethiopia and in Malawi on this study to inform the national vaccine programmes in each country and to ensure the reach of the new vaccines was quickly maximized. Beyond Ethiopia and Malawi, our findings provide critical evidence for practitioners worldwide planning NVIs and for their suppliers on how the composition of demand can impact launch dynamics including forecasting, stock management, service delivery and drop-out rates.

## Methods

The study population included children receiving publicly available new and routine immunizations in Ethiopia and Malawi in 2011–13. Uptake of new vaccines was compared with pentavalent vaccine (Penta), which was already established in the routine immunization system and targets the same age group and immunization schedule.

### Data extraction

Health facility-based data were collected in Ethiopia on three doses of PCV (PCV1, PCV2 and PCV3), and three doses of Penta (Penta1, Penta2 and Penta3) by obtaining vaccination records of all children after PCV launch (October 2011). Data were extracted for all children who were registered in the health facility child immunization registration book as vaccinated within 1 year after PCV launch. Records in Ethiopia included an individual identifier that allowed for tracking vaccinations received for a single child over time.

In Malawi, data were extracted from health facility registers on the same vaccines as well as both doses of Rota (Rota1 and Rota2) for two time periods: 1 year post-PCV launch (November 2011–October 2012) and then again for 6 months post-Rota launch (October 2012–March 2013). Each immunization is recorded on one line of the facility register with date of vaccination and the birthdate of the receiving child. These records were captured for all days within the two time periods. It was not possible in Malawi to track vaccinations received by a single child over time, as this is not recorded. Our data are therefore cross-sectional in nature.

To ensure data were collected from a representative sample of health facilities, a multi-stage cluster sampling approach was used to independently sample health facilities from within Ethiopia and Malawi. Sample size calculations were performed to estimate a precise point estimate of vaccine coverage. In Ethiopia, sampling occurred throughout five regions based on sample size calculations (estimated 32 000 records from 102 facilities) with a confidence level of 95%, precision of 5% and a design effect of three due to the multi-stage cluster sampling method. Within Malawi, sampling occurred within three regions based on sample size calculations (409 440 vaccination records from 42 facilities) with a confidence level of 95% and precision of 5%.

Within Ethiopia, six data collectors extracted data from registration books into data extraction books that were prepared for each health facility. The registration books were scanned and the data collector submitted scanned images of the registration book and the data extraction book to the health centre at the end of the month. The data were double entered using Microsoft Excel and EPIinfo6-D. Within Malawi, six data collectors photographed vaccination records from paper-based child health registers. Data from photographs were double entered in Microsoft Excel or CSPro and exported to a customized Excel database.

### Data

Within Ethiopia, data collection began in January 2012 and was completed in February 2013. Overall, a total of 102 health facilities were sampled to obtain the sample size of 1152 children. Within Malawi, data were collected at 41 facilities from January 2012 to April 2013 for PCV and April 2013 to January 2014 for Rota. During the data collection period, the catchment area of one of the 42 sampled health facilities in Malawi was consumed by another facility included in this study.

Total administrations of the first dose of PCV (PCV1) and Rota (Rota1) were used as the main indicators of demand for the new vaccines. Children receiving vaccinations were categorized into the new birth cohort or backlog cohort based on their age at PCV1 and Rota1 launch. New birth cohort children were those who were eligible to start their routine immunization schedule as of the launch date (6 weeks old) or born any point after. Backlog cohort children receiving PCV1 were those who were older than 6 weeks but less than 1 year of age at the time of introduction. The small group of children who had no birthdate recorded were categorized into an ‘unknown’ group and children who were ineligible when they received the vaccination were categorized as ‘ineligible’; both of these categories were combined to create the cohort of ‘other’ children who received vaccinations. Due to more restrictive age eligibility requirements for Rota1 vaccination (up to 15 weeks instead of 1 year), in Malawi, the backlog cohort consisted of all children aged 6–15 weeks on the date of launch.

To describe the pattern of demand, several key factors were characterized from the collected data. The months since launch were categorized based on the launch date for the facility in Ethiopia and on the national launch dates for PCV and Rota in Malawi. To characterize the number of people seen in any given day per facility regardless of vaccine type, the total number of children who received at least one vaccination per day per facility type was calculated. In Ethiopia, to characterize drop-out rates (proportion of children receiving PCV1 but not PCV3), children were categorized by their completion of the PCV series among all who obtained their first dose of PCV. The data collected from Malawi did not follow the same child for all three doses, and therefore, drop-out rates were not calculated.

Both Ethiopia and Malawi vaccination records were weighted to account for unequal probability of selection and to obtain national-level estimates; for the few purposefully picked facilities, certainty units were created (Potter *et al.* 2003; Pitblado 2009; Thompson 2012). The Central Statistic Agency (CSA) population for 2012 were used for the Ethiopian weights whereas Malawi weights were calculated using the population projections from the National Statistical Office 2008 census, as captured in the 2011 cold chain inventory assessment.

### Analysis

To describe the relative demand for PCV1 and Rota1, the monthly doses of vaccines administered in the post-launch period were estimated and compared with the monthly doses of Penta1 (i.e. the first dose of Penta). Both the relative percent as well as the difference of means *t*-test comparison were calculated for the estimated population values. Penta was used as the benchmark in vaccine uptake as it was a well-established, routine immunization in both countries and its administration shares the same schedule as PCV for all three doses, and with Rota for the first two doses.

The relative composition of the demand of PCV1 and Rota1 was then examined by calculating the proportion and 95% confidence interval for the backlog demand over the total number of doses administered (i.e. the backlog cohort, new birth cohort and other cohort doses). To compare the differences in composition with respect to different age restrictions, the relative composition of demand was compared between PCV1 and Rota1 within Malawi for the same time periods by calculating the proportion of demand attributed to the backlog and the respective 95% confidence interval.

To inform EPI stock planning, we also calculated a planning ratio, which is the ratio of doses for PCV administered to the backlog cohort, ineligible children and children with unknown age vs the new birth cohort. Both the relative percent as well as the difference of means *t*-test comparison were calculated for the estimated population values. Finally, to more thoroughly describe the age at which PCV1 and Rota1 were administered, age histograms were created for the backlog cohort and new birth cohort of children. The proportion of children receiving vaccines after 14 weeks was compared between the new birth and backlog groups using 95% confidence interval estimation.

We then examined the possible implications of the magnitude of the total demand, by plotting the vaccinations administered per day per facility type over time, stratified by facility catchment size. We plotted the daily average facility size against the mean over month since launch to examine trends over time. Additionally, we illustrated the percent of children dropping out during the PCV series according to their age at vaccination within the backlog cohort.

To account for the sampling design in variance estimations, a stratified multi-stage cluster method was declared using Stata 12.

## Results

**Figure 1.**
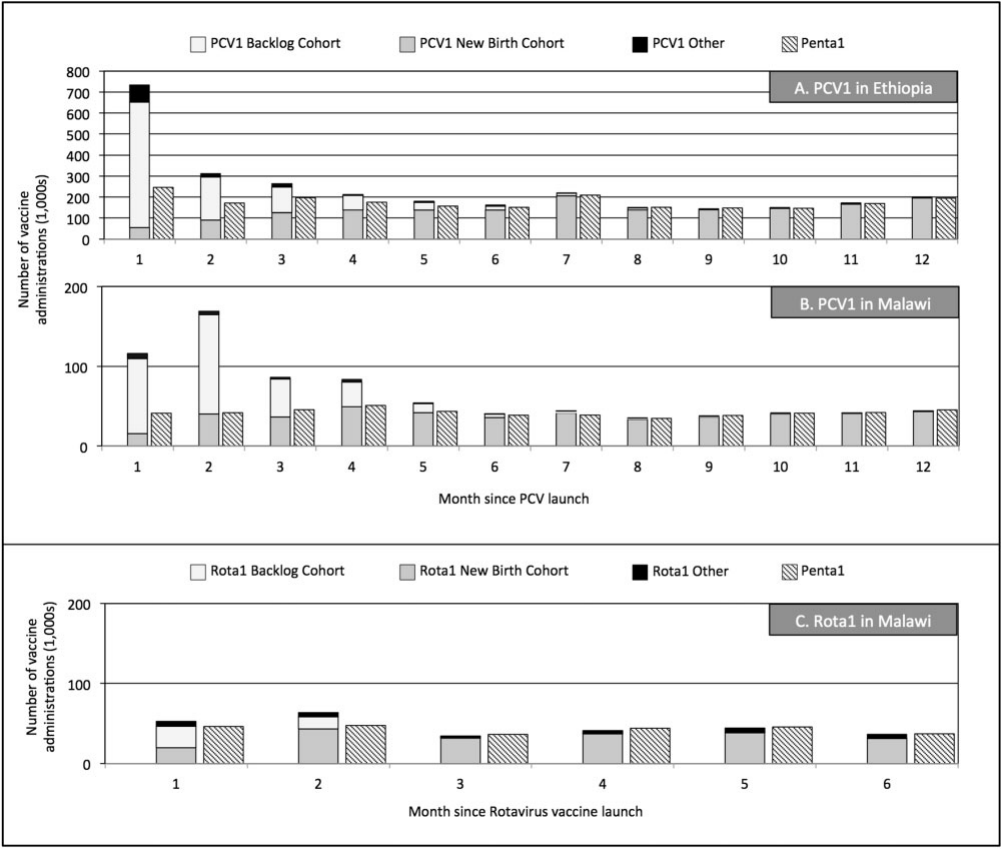
Total number of first doses of PCV and Penta within Ethiopia as well as first doses of PCV, Rota and Penta within Malawi in the months after introduction. (a) PCV1 demand in Ethiopia. (b) PCV1 demand in Malawi. (c) Rotavirus vaccine demand in Malawi

The estimates of the number of first doses of PCV and Rota administered post-launch as well as the composition of this demand are displayed in Figure 1.

In the first year, the relative demand for PCV1 [290.4 per 10 000 (SE 9.4)] compared with Penta1 [212.1 per 10 000 (SE 6.5), *t*-test *P*-value < 0.01] was 37% greater in Ethiopia. In Malawi, the 1-year relative demand for PCV1 [80.1 per 10 000 (SE 0.3)] compared with Penta1 [50.3 per 10 000 (SE 0.6), *t*-test *P* < 0.01] was 59% greater; for the first 6 months, the relative demand of Rota1 [27.2 per 10 000 (SE 0.4)] was 5.9% greater than Penta1 demand [25.7 per 10 000 (SE 0.5); *t*-test *P* < 0.01].

During Months 1–3 post-launch, the demand for PCV1 [130.9 per 10 000 (SE 5.0)] was 112.8% higher than that of Penta1 [61.5 per 10 000 (SE 2.2), *t*-test *P* < 0.01] in Ethiopia; PCV1 [37.2 per 10 000 (SE 0.6)] was 188.8% higher than Penta1 [12.9 per 10 000 (SE 0.1), *t*-test *P* < 0.01] in Malawi; and Rota1 [15.0 per 10 000 (SE 0.1)] was 15.4% higher than Penta1 [13.0 per 10 000 (SE 0.1), *t*-test *P* < 0.01] in Malawi. In comparison, during Months 10–12 post-launch, demand for PCV1 [52.2 per 10 000 (SE 1.7)] vs Penta1 [51.2 per 10 000 (SE 1.6), *t*-test *P* < 0.01] was 2.1% higher than Penta1 in Ethiopia and 1.1% higher in Malawi [PCV1: 12.9 per 100 000 (SE 0.2) vs Penta1: 12.9 per 100 000 (SE 0.3), *t*-test *P* = 0.04]; Rota1 [12.2 per 10 000 (SE 0.3)] demand was 4% lower than Penta1 [12.7 per 10 000 (SE 0.4), *t*-test *P* < 0.01] demand in Malawi for Months 4–6 post-launch.

The consumption of Penta1 over the time of introduction does not drop; instead, the number of children receiving Penta1 increased from the monthly average during the first 3 months after launch by 16% in Ethiopia, 2% in Malawi and 1.3% in Malawi after Rota launch.

The backlog cohort accounted for 37.1% (95% CI: 35.2%, 39.9%) of the total PCV1 demand within the first 12 months in Ethiopia, and 40.0% (95% CI: 37.8%, 42.2%) within Malawi. During Months 1–3 post-launch, the backlog cohort represented the majority of the PCV1 demand for the first 3 months in both Ethiopia (70.7%; 95% CI: 66.9%, 74.1%) and Malawi (71.5%; 95% CI: 70.9%, 72.1%). In comparison, at the end of the post-launch period (Months 10–12) the PCV1 backlog cohort represented a small fraction of the total PCV1 demand in Ethiopia (1.2%; 95% CI: 0.9%, 1.5%) and Malawi (1.1%; 95% CI: 1.0%, 1.2%).

Within Malawi, the backlog cohort accounted for different proportions for the Rota1 and PCV1 vaccine demand. In the 6 months post-launch, the backlog cohort accounted for 16.3% (95% CI: 15.6%, 17.0%) of the Rota1 demand and 56.7% (95% CI: 55.2%, 58.3%) of PCV1 demand. For the first 3 months of Rota1 launch the backlog cohort represented only 28.4% (95% CI: 27.6%, 29.2%) of the total demand, which was significantly different compared with the 71.5% (95% CI: 70.1%, 72.1%) of total PCV1 demand in Malawi.

Table 1 reports the planning ratio, which examines the ratio of all vaccines administered (exclusive of the new birth cohort) compared with the new birth cohort for Ethiopia and Malawi. In the first 3 months, the backlog/ineligible/unknown cohort demand for PCV1 [103.8 per 10 000 (SE 4.2)] was 3.8 times larger than the new birth cohort demand [27.2 per 10 000 (SE 1.2), *t*-test *P* < 0.01] in Ethiopia and 3.0 times higher in Malawi [backlog: 28.0 per 10 000 (SE 0.5) vs new birth: 9.3 per 10 000 (SE 0.09), *t*-test *P* < 0.01]. This drops in later months similarly between the two countries with the final 3 month’s ‘non-new birth cohort’ demand [1.2 per 10 000 (SE 0.1)] 2.4% the size of the new birth cohort [51.1 per 10 000 (SE 1.7), *t*-test *P* < 0.01] in Ethiopia and 2.4% in Malawi [backlog, unknown, and ineligible: 0.5 per 10 000 (SE < 0.01) vs new birth: 12.4 per 10 000 (SE 0.2) *t*-test *P* < 0.01]. The ratio of backlog/ineligible/unknown administrations [7.0 per 10 000 (SE 0.01)] to new birth cohort administrations [20.2 per 10 000 (SE 0.1) *t*-test *P* < 0.01] was 35.0% for the Rota vaccine over the first 6 months after launch.

**Table 1.** Planning ratios for the first dose of PCV in Ethiopia and Malawi and Rota in Malawi

	Ethiopia PCV excluding new birth cohort/new birth (ratio)	Malawi PCV excluding new birth cohort/new birth (ratio)	Malawi Rota (restricted age eligibility) excluding new birth cohort/new birth (ratio)
Months 1–12	0.6	0.7	0.3^a^
Month			
1–3	3.8	3.0	0.6
4–6	0.3	0.4	0.1
7–9	0.1	0.1	n/a
10–12	0.02	0.02	n/a

Note: Ratios are total demand excluding the new birth cohort over the new birth cohort.

^a^ The number only includes the first 6 months of post-launch.

**Figure 2.**
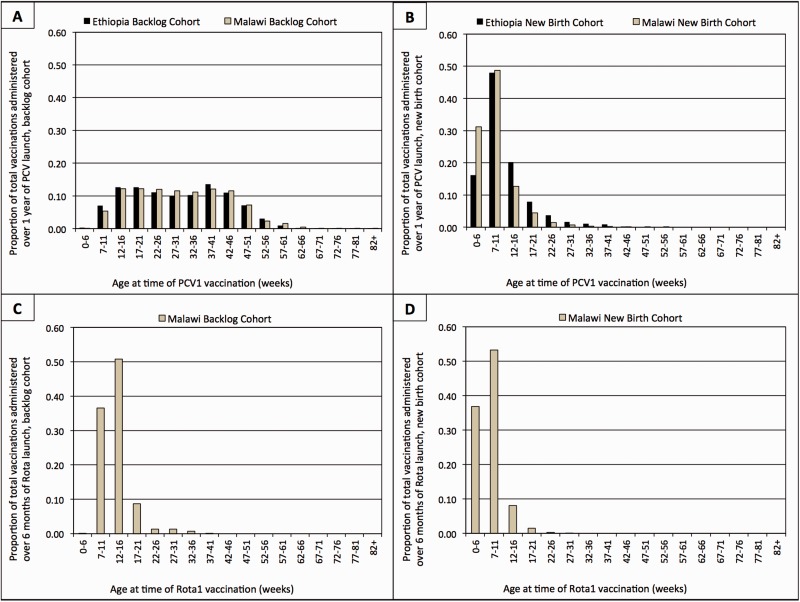
Age histogram for PCV1 and Rota1 backlog cohort vs new birth cohort, Ethiopia and Malawi. (a) Age histogram for PCV1 backlog cohort in Ethiopia and Malawi. (b) Age distribution of PCV1 new birth cohort in Ethiopia and Malawi. (c) Age distribution of Rota1 backlog cohort in Malawi. (d) Age distribution of Rota1 new birth cohort in Malawi.

Figure 2 depicts the age distribution of the new birth and backlog cohort for the first dose of PCV in Ethiopia and Malawi over 1 year and rotavirus vaccine in Malawi over 6 months. As expected, children in the backlog cohort tend to be older when receiving PCV1. 86.7% (95% CI 85.6%, 87.7%) of all backlog cohort PCV1 vaccinations in Ethiopia and 87.5% (95% CI: 86.2%, 88.7%) in Malawi were given after the third dose of PCV is scheduled for administration (>14 weeks), as opposed to just 22.8% (95% CI: 21.3%, 24.3%) of children in the new birth cohort in Ethiopia and of 10.6% (95% CI: 10.2%, 11.1%) children in the new birth cohort in Malawi. The age distribution for children receiving Rota1 in the backlog cohort in Malawi appears less skewed given more restrictive age eligibility for this vaccine.

**Figure 3.**
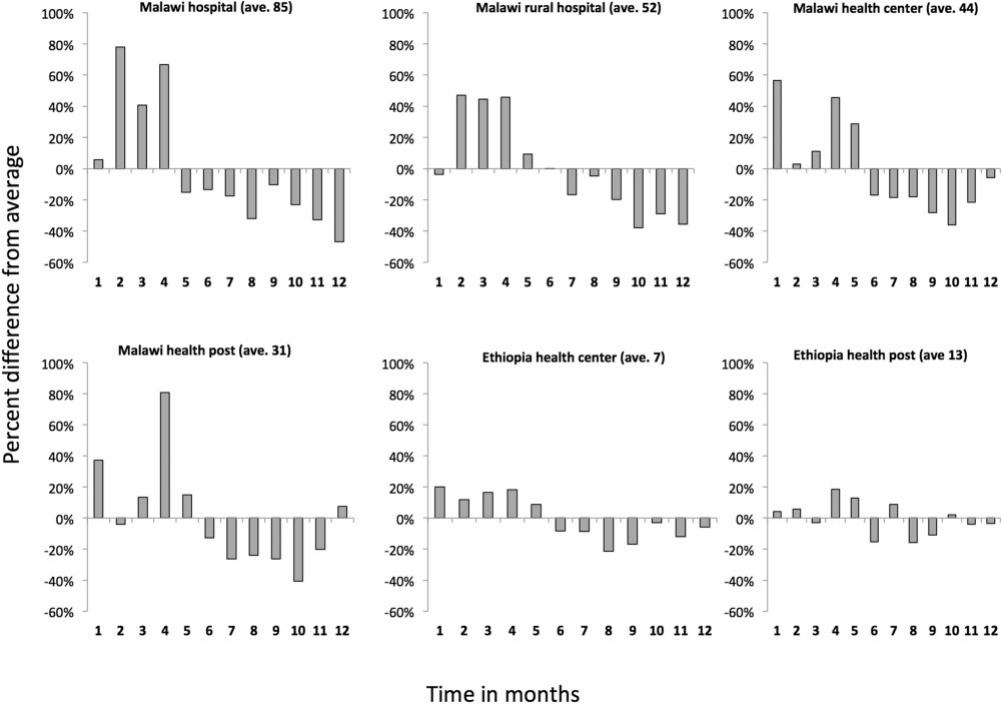
Percent increase in daily vaccinations administered per day by health facility type in the 12 months post-PCV launch

Figure 3 illustrates the percent difference in the daily immunizations provided per day after PCV launch for Ethiopia and Malawi according to different facility types. In general, the immunization session sizes were greater in the first few months after NVI. In the first month post-PCV launch, Malawi health centres were the largest (56.7% higher), in the second month Malawi hospitals (78% higher) and Malawi rural hospitals in the third month (44% higher). The largest increase in Ethiopia was in the first month for health centres (20% higher).

**Figure 4.**
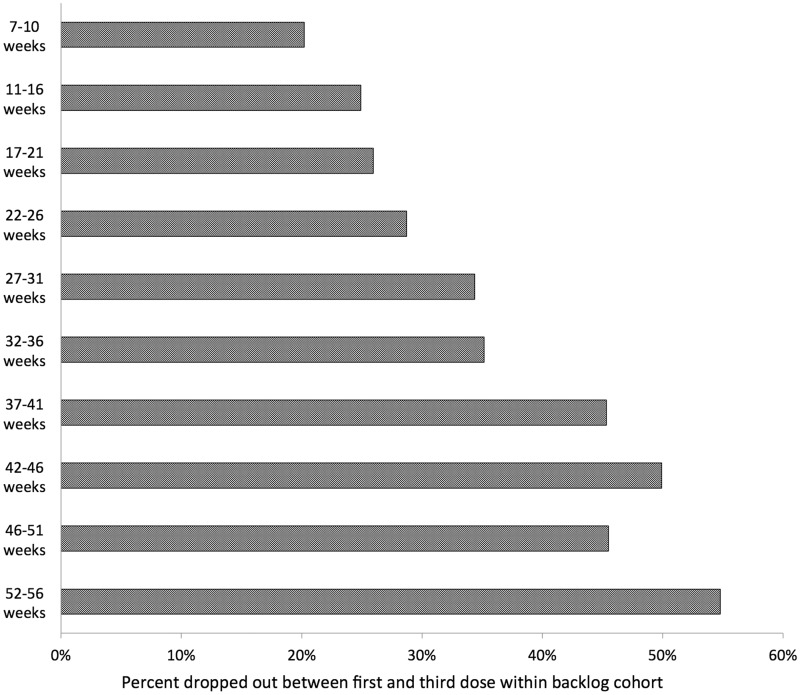
Percent of backlog children who dropped out between the first dose of PCV and third (last) dose of PCV according to age at vaccination in Ethiopia

Figure 4 reports the percent of backlog children that dropped out between PCV1 and PCV3 for Ethiopia. Among older backlog children aged between 37 and 51 weeks old when first vaccinated, the percent that dropped out was between 45% and 50%. In comparison, among younger backlog children between the ages of 7 and 21 weeks old when vaccinated, only 20–26% dropped out.

## Discussion

### Key characteristics of demand

#### Temporality of demand

The Ethiopia and Malawi experiences highlighted that demand for the newly introduced PCV and Rota was greater than for an established routine vaccine (Penta) in the year of introduction. Importantly, we found that this demand was particularly large in the first few months post-launch. A surge in uptake for NVI has been reported in the USA (Lorick *et al.* 2009; Schuck-Paim *et al.* 2013), and Burchett *et al.* (2012) report on frequent stock-outs of new vaccines at the time of introduction, which created a perception that all vaccines were out of stock in the facility. But this is the first documented report quantifying the early surge in uptake for a new vaccine within African countries and across vaccine types.

This work found that the backlog cohort heavily shaped the pattern of demand observed in the first year. Although the WHO guidelines recognize the importance of two birth cohorts (i.e. the new birth and those still eligible to receive vaccinations based on age restrictions) when planning stock levels (WHO 2014a), the information on monthly uptake and relative composition of these two birth cohorts is limited. Although previous guidance suggests that the stock should be double that of the new birth cohort if all children under 1 are considered for the vaccine, our work adds pivotal information that the demand from ‘backlog’ children receiving vaccines was not constant throughout the year. Demand was over three times greater than the new birth cohort in the first 3 months, but this excess demand tapered off by approximately Month 7. Additionally, we found that the relative demand and its composition were similar across the two countries for the same vaccine (PCV), suggesting that the impact of geography on uptake dynamics is limited.

#### Influence of age restrictions

Conversely, we found evidence that uptake dynamics were affected by the age restrictions of the introduced vaccine. Specifically, the age restriction for the first dose of Rota1 was narrow (6–15 weeks) in Malawi when compared with that of PCV1 (any child 6 weeks–12 months old). This difference in age restrictions corresponded with unique patterns of demand. In the first 3 months post-Rota launch, the backlog demand for Rota was small—only 28.4% of the total demand, as compared with 71.5% for the PCV introduction. As a result, the excess demand for Rota as compared with the benchmark vaccine was only 15.4% higher.

Although this smaller demand for Rota could be due to lesser social mobilization efforts rather than differing age restrictions, we observed that this was not the case. In fact, additional health care worker trainings and social mobilization activities were planned for the Rota vaccine launch compared with the PCV launch. Further, a study on caregiver knowledge, attitudes and practices conducted prior to the rotavirus vaccine launch found a high level of desirability, acceptability and interest in Rota, suggesting that caregivers did indeed wish to vaccinate their children (Munthali and Mvula 2012). Thus, the likely driver of the difference in the uptake dynamics is not social mobilization but age restrictions, which inherently limit the size of the backlog cohort. Therefore, although the backlog demand is always an important factor when considering any vaccine launch, the findings suggest that its relative size is shaped by the age restrictions.

#### Impact on routine immunization

Lin *et al.* (2005) identified that in the USA the introduction of PCV created some disruptions in the timely delivery of other vaccines. Burchett *et al.* (2014)’s six case studies conducted in LMICs found that although the NVIs were viewed as intrinsically positive, on the whole there was no evidence that they had any major impact, positive or negative, on the broader health system. Hyde *et al.* (2012)’s review of the literature also found that vaccines did not impact the coverage of previously introduced vaccines. Our study identified slightly more positive effects of NVIs on routine immunization systems in Ethiopia and Malawi. We observed that the addition of the new vaccine to the immunization schedule did not negatively impact Penta1 levels as illustrated in Figure 1. In fact, Penta1 had a higher than average administration in the first 3 months after both PCV introductions, at the same time as the PCV demand was the strongest. This suggests that additional children, mobilized by the introduction of PCV1, received their needed routine vaccinations.

#### Age at vaccination and drop-out

We found that the backlog cohorts for PCV1 in both Ethiopia and Malawi were comprised mainly of children over the age of 14 weeks. This finding highlights a potentially at-risk group of children who may not receive all of the three doses of PCV, as these older children do not have another scheduled vaccine dose to receive until 9 months of age (first dose of the measles vaccine). Because children must return to clinics ‘off schedule’ just to receive the full PCV dosing, they may be at greater risk of dropping out.

### Policy and programme management implications

These characteristics of demand composition should be taken into account when planning NVIs. Forecasting, stock planning, service delivery, reducing drop-out and monitoring can only be adequate by integrating strategies that rest on solid understanding of the backlog cohort’s demand.

#### Forecasting and setting age eligibility for a new vaccine

Countries with inclusive age eligibility criteria should request volumes of commodities (vaccines, injection materials, safety boxes, etc.) from suppliers based on forecast estimates that include the backlog cohort’s demand, if required. In addition, WHO should provide clearer recommendations to countries on the inclusion of the backlog cohort in their demand forecasts. This would allow donors such as Gavi, to revise their application materials to include more explicitly a forecast for the backlog cohort.

Here we have seen that with PCV introduction in Ethiopia and Malawi, 37% and 59% more new vaccine doses were required compared with the benchmark routine vaccine (Penta) over the course of 1 year, primarily due to the backlog. For Rota in Malawi, only 5.9% more doses were required than for the routine vaccine over the first 6 months, likely because the age restrictions for the new vaccine were stricter.

Thus, when setting age eligibility threshold for a new vaccine, countries should consider the impact of these guidelines on the size of the backlog cohort demand. Widening age eligibility on a new vaccine will lead to an increase in total demand for the vaccine (up to ∼60% more for PCV over the first year). Setting stricter age eligibility rules is an option to limit the financial and operational burden of NVI, but immunizing the backlog cohort is a significant opportunity to reach more children and accelerate herd immunity where epidemiologically relevant.

#### Stock planning

If inclusive age eligibility restrictions are set, a large buffer stock for the new vaccine should be available beginning on the day of launch. Because demand from the backlog cohort is higher in the first months, failure to preposition additional stock would result in stock-outs.

In Ethiopia and Malawi, excess demand outside the new birth cohort (including backlog, ineligible/unknown demand) for the new PCV was, respectively, 3.8 times and 3.0 times that of the new birth cohort in the first 3 months post-introduction. This ratio rapidly decreased, and excess demand was minimal by Month 7 in both Ethiopia and Malawi. The best practice is thus to preposition new vaccines at sub-national levels, e.g. district and facility levels, in the first months of introduction beyond the routine monthly allocation (up to four times more stock) ahead of launch. This may require putting in place temporary coping mechanisms if the cold chain capacity is restricted (e.g. a more frequent vaccine distribution schedule). Delivery schedules should align with the need to preposition stock, and contingency plans should also be put in place to allow for the allocation of transportation in the event of required emergency stock deliveries.

#### Service delivery and daily vaccinations administered

Countries that intend to launch new vaccines with broader age eligibilty rules should prepare for increased service delivery needs at the time of introduction. Indeed, children who have already received routine vaccines come to the facilities at the same time as the new birth cohort. In Ethiopia and Malawi, immunization sessions were larger during the first month of PCV introduction: the average daily number of immunizations provided increased by 20% and 56% in Ethiopian health centres and Malawian hospitals, respectively. The average number of vaccinations provided remained higher than normal during the first 3 months of introduction, due to continued demand from the backlog cohort. Rota was introduced with narrower age restrictions, had a lower excess demand from the backlog cohort, and therefore, did not appear to have an impact on the average daily number of immunizations provided as observed at health facilities. Labour cost accounts for an important share of total costs for immunization. In their study on costing and financing of routine immunization and new vaccines (EPIC) in six countries, Brenzel *et al.* (2015) estimated that the value of labour time ranged from 19% (Benin) to 65% (Moldova) of immunization costs. But there is little agreement in the literature on whether NVI requires an increase in the size of the health care workforce, or whether the additional demand can be absorbed by existing health care workers. Hyde *et al.* (2012) report from their literature review that the impact of NVI was variable, but that it was limited for vaccines introduced into the already existing immunization schedule. In Burchett *et al.*’s (2012) survey, 61% of health facility respondents in six countries reported that workload had increased at the time of, or just after, the NVI. The authors mention this could be due to a temporary ‘catch-up’ vaccination of older children. However, they report no change in staffing numbers or distribution as this increased workload was absorbed by existing workers. Usuf *et al.* (2014) also find that Gambian staff were not recruited specifically for the PCV introduction, presumably because the work was absorbed within existing slack—thus labour was not part of the incremental cost of NVI. However, Lydon *et al.* (2014) point to a global shortage of health workers worldwide, particularly in low- and lower-middle-income countries. They project that ‘substantial increases in full-time equivalent staff for vaccination’ will represent the better part of non-vaccine health system investments over the next decade, in the context of frequent NVIs. Shen *et al.* (2014) also stress that the growing complexity of immunization programmes increases the need for a well-trained, capable health workforce.

For future introductions with inclusive age restrictions, policy-makers should be mindful that excess demand from the backlog cohort may strain the health workforce, particularly in hospitals. Countries should thus carefully estimate whether the introduction of a new vaccine requires increasing human resource capacity at the health facility level during the first months following NVI, or whether the added workload can be absorbed by existing capacity. The need for increasing facility staff, which could also be a consequence of the widening of age eligibility criteria advocated in this manuscript, could negatively impact the cost-effectiveness of the new vaccines.

#### Preventing drop-outs

Policy-makers planning future introductions must pay particular attention to the higher risk of drop-out of the backlog cohort. ‘Backlog’ children are out of sync with the official routine immunization schedule for the new vaccine. These children will require a greater number of visits to the health facilities to be fully immunized, as they typically do not benefit from the co-administration of the new vaccine for their second or third visit.

We observed in Ethiopia that the drop-out rate for older children was higher than for younger children: over 35% of children aged 32 weeks or above did not complete the PCV schedule, as opposed to 20% of children aged 7–10 weeks. For future introductions, service delivery and social mobilization activities must mitigate this drop-out risk. Health care workers must be taught during pre-introduction training how to emphasize to caregivers of older children the need to come back to the health facility for additional visits. Social mobilization activities should also clearly underscore the number of doses required to be fully immunized, as well as clearly highlight age eligibility to reach children in the backlog cohort.

#### Monitoring stock levels

Countries introducing new vaccines must update their data tools well ahead of the launch to ensure proper tracking of new vaccine delivery and consumption. After launch, countries should frequently monitor stock levels at the regional and district levels in the first 3–6 months. They should be ready to respond with emergency orders as needed.

The Ethiopia and Malawi experiences highlight the need to closely monitor uptake dynamics to ensure against vaccine stock-outs, and to help the new vaccine quickly reach routine immunization coverage levels. In Malawi, this was done both via targeted phone calls to health facilities every 2 weeks and in-person supportive supervision visits. Due to the large backlog demand observed over the first months, this stock monitoring was essential to identify the need for additional stock in a timely fashion.

More generally, replicating uptake studies such as the one we performed in Ethiopia and in Malawi offers an opportunity for policy-makers to better understand the profile of their countries’ demand for immunization, and thus better plan for the next introduction. Indeed, launching a vaccine should not be seen as an event but as a process, and the appropriateness of plans (regarding forecasting, stock management, service delivery) can only be determined during the first year of introduction.

Beyond vaccines, similar ‘backlog’ patterns are observed in other health areas that deal with age cohorts, such as guideline changes on CD4 count that impact viral load testing eligibility for HIV infection. For these areas, similar studies assessing uptake dynamics of new life-saving products would allow practitioners to better plan for their introductions, and thus maximize the opportunities they offer.

## Conclusion

The WHO’s Decade of Vaccines Collaboration Research and Development Working Group has outlined the need for targeted implementation research to improve the uptake of new vaccines (Arora *et al.* 2013). CHAI has worked with low- and middle-income countries on 10 NVIs, and derived from its experiences a best practice toolkit for practitioners (CHAI 2014). In particular, through the analysis of the composition of demand for recent vaccine introductions in Ethiopia and Malawi, important recommendations can be drawn for future practice.

When planning an NVI, countries should consider the impact of the backlog cohort, especially in the first 6 months of introduction. Countries can maximize the life-saving potential of immunization through vaccinating a larger share of the backlog cohort, by setting looser age regulations and engaging in significant social mobilization. To cater to this backlog cohort, countries’ plans should request high stock volumes for the new vaccines, allocate adequate human resources and cold chain capacity to accommodate backlog cohort demand and include increased social mobilization activities to reach more children and reduce drop-out.
